# *Eurycoma longifolia*—Infused Coffee—An Oral Toxicity Study

**DOI:** 10.3390/nu12103125

**Published:** 2020-10-13

**Authors:** Norzahirah Ahmad, Bee Ping Teh, Siti Zaleha Halim, Nor Azlina Zolkifli, Nurulfariza Ramli, Hussin Muhammad

**Affiliations:** Herbal Medicine Research Centre, Institute for Medical Research, National Institutes of Health, Ministry of Health Malaysia, Setia Alam, Shah Alam 40170, Malaysia; bpteh_km@yahoo.com (B.P.T.); ctzaleha.h2@gmail.com (S.Z.H.); azlina.zolkifli@moh.gov.my (N.A.Z.); nurulfariza.r@moh.gov.my (N.R.); hussin.m@moh.gov.my (H.M.)

**Keywords:** *Eurycoma longifolia*, Tongkat ali, toxicity, infused coffee, herbal additives

## Abstract

Coffee infused with the additive *Eurycoma* *longifolia*, also known as Tongkat ali (TA), has become widely available in the Malaysian market. Safety evaluations for consumption of the products have been called for due to the herbal addition. This study investigates the acute, subacute and chronic effects of a commercial TA coffee in Sprague Dawley rats when given in a single, repeated and prolonged dosage. The dosages of 0.005, 0.05, 0.30 and 2 g/kg body weight (BW) were used in the acute study and 0.14, 0.29 and 1 g/kg BW were used in the repeated dose studies. The in-life parameters measured were food and water intake, body weight and clinical observations. Blood were collected for hematology and clinical biochemistry analyses. All animals were subjected to full necropsies. Non-toxicity-related changes were observed in the food and water consumption parameters. Body weight showed normal increments and none of the animals had any clinical signs of toxicity. Microscopically assessed organ tissues did not reveal any abnormalities. There was significant decrease of platelet count in all the chronic study male treated groups. Significant elevation of renal profile parameters in both gender groups given 0.29 g/kg BW, along with liver and lipid profile elevation in some female groups of the chronic study were noted. No dose-dependent relationship was apparent in the dosage range tested, though these changes may suggest an initial safety indication to the TA coffee. The study concludes that the no observed adverse effect level (NOAEL) for this commercial TA coffee was 1 g/kg BW.

## 1. Introduction

*Eurycoma longifolia,* also known as Tongkat ali (TA), is a native plant to Southeast Asian rain forests. The roots are believed to boost wellness while having aphrodisiac, anti-malarial and other therapeutic properties, such as anti-inflammatory and anti-osteoporosis [1,2,3,4,5,6,7,8]. TA has received considerable attention among Malaysian consumers and is traditionally processed into a drink, although coffee makers also tout it as a healthy additive in coffee drinks. Safety information such as the no observed adverse effect level (NOAEL) for *E. longifolia* in its aqueous extract form has been previously reported to be more than 3 g/kg in mice [9] and more than 1 g/kg and 5 g/kg in rats in two separate studies [10,11], while the powdered form of the root was reported to have an acute limit dose of more than 6 g/kg [12]. Although these studies reported a high tolerance of the highest dose used, they also reported hydropic liver histology indicating hepatotoxicity [11] and minor yet significant hematology and clinical biochemistry changes [10,12]. Additionally, information for TA extract long-term consumption is limited and its use as an herbal additive in food products has never been evaluated and warrants further investigation.

Worldwide, the legislation with regards to beverages with herbal additives varies between countries [13], whereas currently in Malaysia, safety testing for food products in this category has not been accounted under the Food Act (1983), Ministry of Health, Malaysia and Food Regulation (1985), Ministry of Health, Malaysia [14,15]. While there have been no claims of ill health and the existing legislation in Malaysia does not require safety testing for using *E. longifolia* extracts in coffee, the World Health Organization (2004) recommends herbal medicinal ingredients in products to be assessed for its safety [16]. Furthermore, the addition of therapeutically claimed traditional ingredients have clouded the definitions between the food and drug regulations. It is often unclear which testing standards are required and the sets of regulations to abide by for the herbal additives in food products [17,18,19]. Consequently, without safety testing, marketing of such coffee outside of Malaysia is restricted due to strict import regulations with various levels of evidence requirements.

Safety testing adds value to the product, elevates the product safety information, improve consumer trust and may prevent potential incidents such as interaction between pharmaceutical drugs and herbal-incorporated food products, where occurrence may have gone unnoticed [17,18,20,21]. The aim of this paper is to investigate the safety of a commercial TA coffee, with reference to the Organization for Economic Cooperation and Development (OECD) testing guidelines 420, 407 and 452 [22,23,24]. The TA coffee was administered to Sprague Dawley rats in a single-dose 14-day acute study and as a repeated daily dose for 28 days and 6 months in the subacute and chronic toxicity studies, respectively.

## 2. Materials and Methods

### 2.1. Test and Reference Item Preparation

The commercial GTHerb Gold Coffee *E. longifolia*-mixed coffee was provided by GTHerb Industries Sdn. Bhd. Analysis of the content was done by Als Technician and Technology Park Malaysia, Biotech Sdn. Bhd. (Kuala Lumpur, Malaysia). The TA coffee (test item) was physically in powdered form and dark brown in color. Prior to the daily preparation, the test item was kept at temperatures between 25 and 28 °C. The test item prepared was calculated based on the individual rat′s body weight and according to the required dosage. The test item for rats weighing less than 100 g was dissolved in 1 mL hot distilled water, whereas for rats weighing 200 g and above, 2 mL of hot distilled water was used. The reference item (distilled water) was administered in the same manner. The administered amount of test item was adjusted weekly to correspond with the weekly change in the body weights.

### 2.2. Care and Handling of Experimental Animal

The three oral toxicity studies used a total of 208 Sprague Dawley rats purchased from BioLASCO Taiwan Co. Ltd. (Taipei City, Taiwan). The rats, hereafter referred to as the test system (TS), arrived at four weeks of age and were quarantined for two weeks under the care of the veterinarian. Following quarantine, the TS were housed individually in the Individual Ventilated Cage (IVC) and acclimatized to the experimental environment for seven days. The room had a 12 h light and dark cycle where the lights were switched on from 7 a.m. to 7 p.m.; the temperature was kept at 22 ± 3 °C and relative humidity was maintained at 57.5 ± 7.5%. The cages and bedding were changed every two weeks. Food (200 g) was placed in each cage weekly and replenished if inadequate. The amount of food left weekly was measured and used to calculate the week’s food consumption. Reverse osmosis water was provided ad libitum and the water intake was also measured in a similar approach. This study followed the Principle and Guide to Ethical Use of Laboratory Animals prepared by the Ministry of Health Malaysia (2000) [25]. Approval from the Animal Care and Use Committee (ACUC) at the Institute for Medical Research, Malaysia was obtained; ACUC number ACUC/KKM/02(5/2008).

### 2.3. Single Dose Acute Oral Toxicity Study

The acute oral toxicity was done following the OECD Guideline No. 420 [22], where the sighting study was done using single dosages of 0.005, 0.05, 0.30 and 2 g/kg BW of test item and administered orally to female TS. First, one of the TS was orally administered with the lowest dose and observed for any signs of acute toxicity 24 h post-dosing. As the first TS showed no signs of toxicity, a second TS was administered the second lowest dose (0.05 g/kg BW) and observed in the same manner. This procedure was repeated until the highest dose (2 g/kg BW) was administered. The highest dose did not trigger any acute effects; thus 2 g/kg BW was used in the main study. Four new TS were given 2 g/kg BW of the test item and monitored for 14 days. The TS were monitored for any signs of acute toxicity, including morbidity and mortality, at 0.5, 1, 2 and 4 h after treatment, then twice daily until day 14. The BW, food and water consumption were measured weekly. The TS were sacrificed following the 14-day observation period and gross observations of all organs were recorded.

### 2.4. Subacute 28-Day and Chronic 6-Month Oral Toxicity Studies

#### 2.4.1. Subacute 28-Day Experimental Design

The subacute oral toxicity study was based on the OECD Guideline No. 407 [23], with modifications. Male and female TS were grouped into four groups of five TS, where each group received doses of 0.14, 0.29 and 1 g/kg BW of test item, orally administered in 2 mL volumes. The control group received distilled water. The TS were observed twice daily for any clinical signs, morbidity and mortality. Individual TS’s dosage was corrected each week to correspond with the TS’s body weight. Detailed clinical observations and food intake measurements were performed, while water was given ad libitum. The TS were anaesthetized and blood samples were collected by cardiac puncture. Blood samples were sent for hematology analysis (2.5 mL) in EDTA tubes. The TS were then sacrificed with an overdose of diethyl ether and necropsies were performed. At necropsy, gross pathology was conducted and the internal organs; lung, heart, liver, kidneys, adrenals, ovaries, testes, spleen, stomach and intestinal tract were collected. All organs were cleaned of excess fat and absolute weights were taken immediately. The organs were then placed in 10% formalin for histopathology evaluations. 

#### 2.4.2. Chronic 6-Month Experimental Design

The chronic 6-month oral toxicity study was carried out in accordance with OECD Guideline No. 452 [24], with modifications, where 20 rats per group per sex were used in the same dosage group (0.14 g/kg BW, 0.29 g/kg BW, 1 g/kg BW and Control) as in the subacute study. The test item (2 mL) was orally administered daily. During the in-life phase, the TS were observed daily for mortality, morbidity and clinical observations. Their food and water intake were monitored weekly, while detailed clinical observations by the attending veterinarian was conducted monthly. At necropsy, gross observation was conducted and blood was collected for hematology and serum clinical biochemistry analysis. The absolute weights of the internal organs were recorded, then treated with 10% formalin for histopathology investigation. The list of organs was as in the subacute study.

#### 2.4.3. Hematology Analysis

Blood was analyzed using Medonic CA620 Vet Analyzer (Boule Diagnostics AB, Stockholm, Sweden) for white blood cells (WBC), red blood cells (RBC), hemoglobin (HGB), hematocrit (HCT), mean corpuscular hemoglobin concentration (MCHC) and platelets (PLT) for the subacute study. In the chronic study, the RBC, HGB, HCT and PLT levels were determined.

#### 2.4.4. Clinical Biochemistry Analysis

Serum in the chronic study was analyzed using the biochemistry analyzer (Vitalab Selectra E-series, Vital Scientific N.V., Spankeren/Dieren, Netherlands). Liver function profile—total protein, albumin, enzymes; alkaline phosphatase (ALP), alanine amino-transferase (ALT) and aspartate amino-transferase (AST), lactate dehydrogenase (LDH), creatine kinase (CK); renal profile—creatinine, urea and uric acid; lipid profile—cholesterol and triglycerides; and glucose and calcium were determined.

#### 2.4.5. Relative Organ Weight

The weight of the organs relative to 100 g BW of the TS were calculated based on the TS body weights recorded prior to necropsy and their absolute internal organ weights.

#### 2.4.6. Histopathological Examination

All organs collected (lung, heart, liver, kidneys, adrenals, ovaries, testes, spleen, stomach and intestinal tract) were fixed in 10% formalin, sectioned and stained using hematoxylin and eosin (H&E) before microscopically observed for any pathological abnormalities. 

### 2.5. Statistical Analysis

The data for body and organ weights, food and water consumptions, as well as hematology and clinical biochemistry results, was checked for normality using Kolmogorov-Smirnov and Shapiro-Wilk tests. Normally distributed data were analyzed using the one-way analysis of variance (ANOVA), while in not normally distributed cases, Kruskal-Wallis test was used. In cases where statistically significant differences were noted, Post-Hoc tests were used to elucidate the differences between the control and treated groups. Male and female data were analyzed separately. When there was any death during the study period, the data for body weight, food intake and water intake were included up until the last recorded weekly data and then excluded thereafter. Consequently, the hematology, clinical biochemistry and organ weight data were excluded for animals that died in the group. The actual sample size is denoted in the result figures and tables. Statistical analyses were performed using SPSS 18.0 statistical software (SPSS Inc., Chicago, IL, USA) and GraphPad Prism 7.0 software (GraphPad Software, La Jolla, CA, USA). Statistically significant differences were considered when *p* < 0.05.

## 3. Results

### 3.1. Single Dose Acute Oral Toxicity Study

No mortality was recorded during the 14-day observation period and the TS showed no sign of toxicity. No major findings were detected in the body weight and food intake parameters of the TS (Table A1). Internal and external examination of the TS and organs revealed no abnormalities. It was concluded that the test item exerted no acute toxicity in the TS. 

### 3.2. Subacute 28-Day and Chronic 6-Month Oral Toxicity Studies

#### 3.2.1. Clinical Signs and Mortality

The TS did not produce any mortality or toxicology-related clinical signs due to the consumption of the test item in either study. However, in the subacute study, one female TS in the medium dosage group on day 18 died due to a gavage accident. While in the chronic study, five females given 0 g/kg BW (day 56), 0.14 g/kg BW (day 121), 0.29 g/kg BW (two TS; day 44 and day 126) and 1 g/kg BW (day 158) and 2 males given 0 g/kg BW (day 158) and 1 g/kg BW (day 76) died also due to gavage accidents. Autopsy revealed no treatment-related alteration in the dead TS.

#### 3.2.2. Body Weights, Food and Water Intake

**Figure 1:**
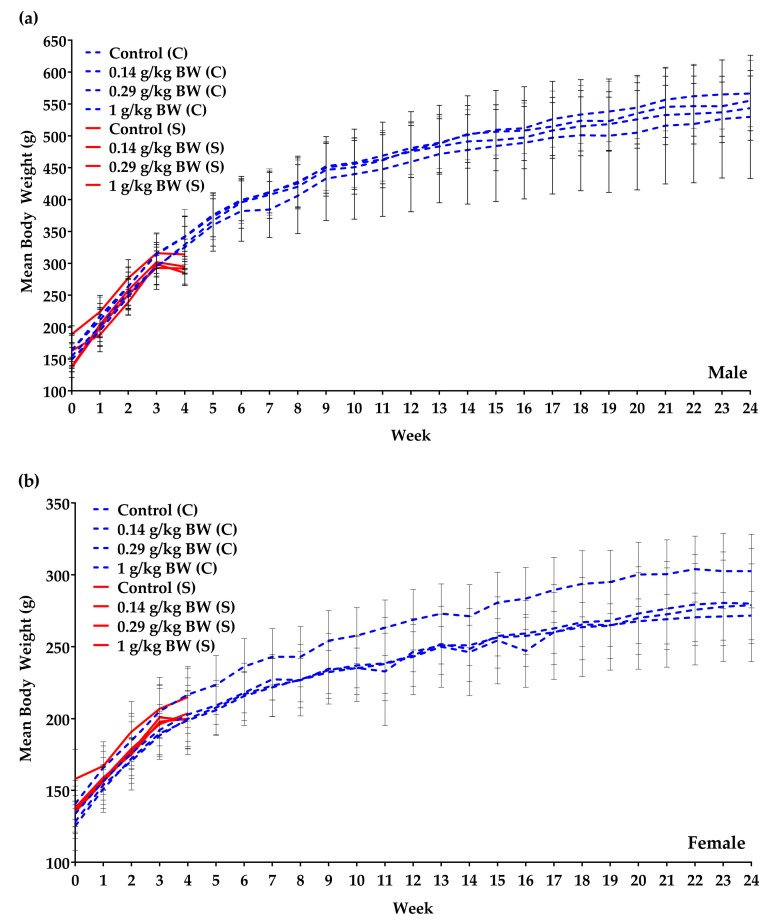

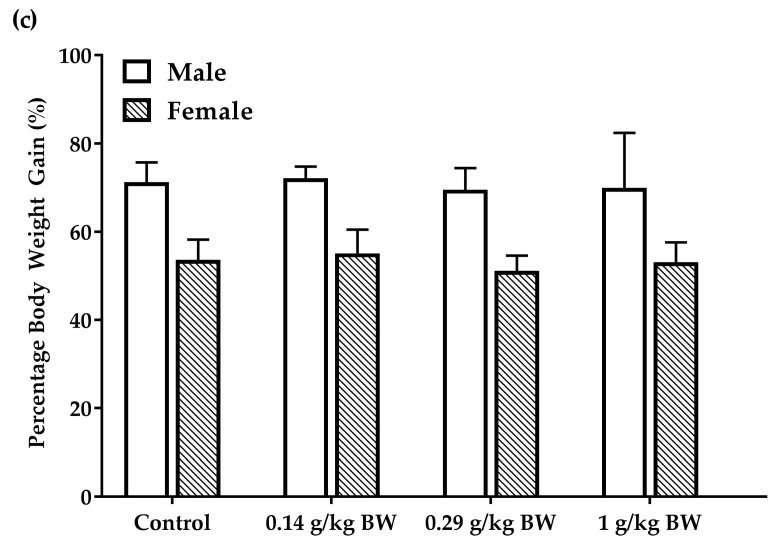
In-life parameters monitored in male and female test systems: (**a**) Mean body weight (BW) of male test systems in the 28-day subacute toxicity study (red line) and the 6-month chronic toxicity study (dotted blue line). (**b**) Mean body weight of female test systems in the 28-day subacute toxicity study (red line) and the 6-month chronic toxicity study (dotted blue line). (**c**) No difference in the body weight (BW) gain in male and female test systems of the 6-month chronic toxicity study. (Group 0.29 g/kg BW female (S): n = 5, week 1–2; n = 4, week 3–4. Group 0 g/kg BW male (C): n = 20, week 1–22; n = 19, week 23–24. Group 1 g/kg BW male (C): n = 20, week 1–11; n = 19, week 12–24. Group 0 g/kg BW female (C): n = 20, week 1–8; n = 19, week 9–24. Group 0.14 g/kg BW female (C): n = 20, week 1–17; n = 19, week 18–24. Group 0.29 g/kg BW female (C): n = 20, week 1–6; n = 19, week 7–18; n = 18, week 19–24. Group 1 g/kg BW female (C): n = 20, week 1–22; n = 19, week 23–24). S = subacute, C = chronic.

The subacute study showed that the mean body weights for both males and females did not depict any significant difference between the control and the treated groups. The weekly mean body weight also increased gradually over the dosing period (Figure 1a,b). The food consumption (Table A2) was noted to be normal for growing TS and no significant increase or decrease was evident.

**Figure 2:**
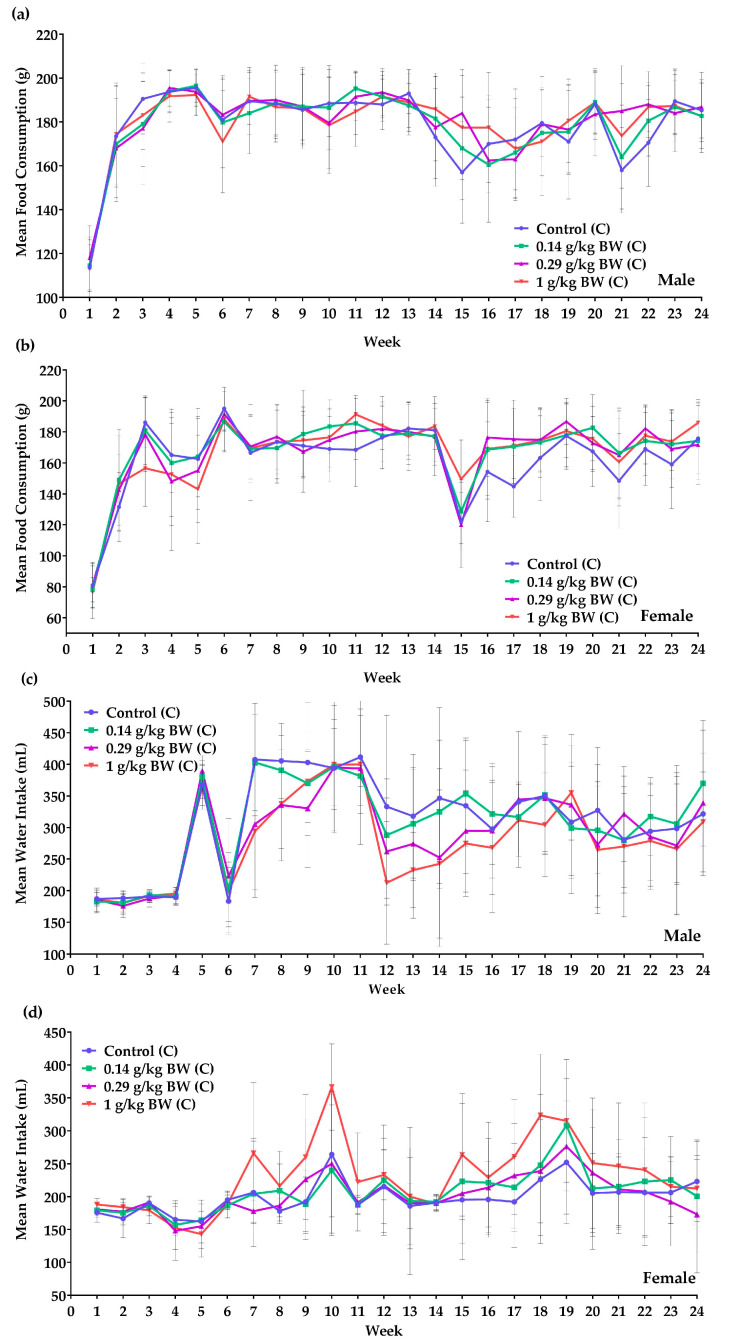
In-life parameters monitored in male and female test systems: (**a**) Mean food consumption of male test systems in the 6-month chronic toxicity study. (**b**) Mean food consumption of female test systems in the 6-month chronic toxicity study. (**c**) Mean water intake of male test systems in the 6-month chronic toxicity study. (**d**) Mean water intake of female test systems in the 6-month chronic toxicity study. Each graph is expressed as mean ± SD (Group 0 g/kg BW male: n = 20, week 1–22; n = 19, week 23–24. Group 1 g/kg BW male: n = 20, week 1–11; n = 19, week 12–24. Group 0 g/kg BW female: n = 20, week 1–8; n = 19, week 9–24. Group 0.14 g/kg BW female: n = 20, week 1–17; n = 19, week 18–24. Group 0.29 g/kg BW female: n = 20, week 1–6; n = 19, week 7–18; n = 18, week 19–24. Group 1 g/kg BW female: n = 20, week 1–22; n = 19, week 23–24).

In the 6 months chronic study, female rats administered 1 g/kg BW dosage of the test item had a significant (*p* < 0.05) increase in body weight each week (Figure 1a,b). However, there was no significant difference in the percentage body weight gain throughout the study of TS administered 1 g/kg BW as compared to the control group in either gender (Figure 1c). Overall, elevating trends in body weight was observed weekly. Looking at food consumption (Figure 2a) in males, a significant increase was observed in weeks 15, 21 and 22 in the 0.29 g/kg BW dosage group when compared to the control group. A significant increase in food intake was seen in females (Figure 2b) given 1 g/kg BW dosage in weeks 11 and 15 when compared to the control group. In week 17, a significant increase in food intake was recorded for all the treated groups. Female TS given 1 g/kg BW showed a significant decrease in food consumption in week 3.

The water consumption trend (Figure 2c) in male TS showed that TS administered 1 g/kg BW consumed less water in weeks 7, 8, 12 and 13, while those given 0.29 g/kg BW only showed a decrease in week 8. Female TS given high doses of the test item (1 g/kg BW dosage) had a significant increase in water intake in weeks 1, 8–10 and 18 (Figure 2d). A significant increase in water intake of female rats given low and medium dosage test item occurred in week 8 and week 22, respectively.

#### 3.2.3. Hematology Analysis

No abnormal levels were detected in the hematological analysis of the blood samples for the subacute study (Table 1). After the 6-month prolonged exposure, the hematologic profile (Table 2) showed a decrease in the platelet (PLT) count in all treated male dosage groups, while other parameters did not show any significant difference. Females given 1 g/kg showed a significant increase in HGB levels when compared to the control group.

#### 3.2.4. Clinical Biochemistry Analysis

The liver and enzyme parameters showed reduced levels of albumin, alkaline phosphatase (ALP), alanine amino-transferase (ALT) and triglyceride in males administered 0.14 g/kg BW (Table 3). Significantly elevated total protein, creatinine and urea levels in males administered 0.29 g/kg BW was observed. There was also a significant increase in creatine kinase level in males given 1.0 g/kg BW dosage. In female test groups, elevated albumin, ALT, aspartate amino-transferase (AST), creatinine, urea, cholesterol and calcium levels were observed in the 0.29 g/kg BW group. The 1 g/kg BW group had a significant increase in lactate dehydrogenase (LDH), cholesterol and calcium levels, while there was a significant decrease in the triglyceride level.

#### 3.2.5. Organ Weights

In the subacute study, there was no significant difference in the relative organ weights (ROW) of any organs in males. Some ROW in females were significantly decreased, including stomachs in the 0.14 g/kg BW group and spleens for the 0.14 and 0.29 g/kg BW groups (Table 4). In the 6-month chronic study, no significant differences in the ROW was observed in any of the collected organs for all the tested groups (Table 5).

#### 3.2.6. Gross Necropsy and Histopathology Examination

No abnormalities were visually detected when gross pathological examination was carried out postmortem. Microscopic histopathology revealed no abnormalities.

## 4. Discussion

Herbal-infused beverages have been introduced into the market where countless other beverages are marketed. With the extra edge of having herbal concoctions incorporated, herbal additives in beverages have gained popularity and are widely consumed in Malaysia. A rat model was used to investigate the acute and prolonged repeated consumption of such product to establish its safety profile and infer its effects at its expected exposure dose in humans.

The test item did not exert any acute toxicity and no mortality was observed at any of the tested doses. In both the subacute and chronic studies, there were no significant clinical signs of toxicity, body weight changes were normal and gross and histopathology investigations did not reveal any significant toxicity effect. There was a total of seven incidental deaths in the repeated dose studies due to technical error by personnel. The gavage error may have led to gavage-assisted reflux in the TS causing these deaths [26]. We report the following findings, namely fluctuations in the food and water intake measurements, the relative organ weights (ROW) in two organs in the subacute study and platelet changes observed in the chronic study with some alterations to the clinical biochemical parameters.

The statistically significant reduction in food consumption which only occurred in the female high dose group in week 3 of the chronic study and the increased water intake in some female treated groups may indicate natural physiological changes. It has been reported that female TS consume less food and become active during their estrus cycle which may incur responses as manifested in the amount of food and water consumed, as seen in this study [27,28]. On the other hand, males in the chronic studies consumed significantly less water, where coffee consumption itself may have caused the TS to drink less water [29]. Despite the fluctuating food and water intake, the body weight measurements showed elevating trends, indicating normal development of the TS. Similar body weight trends were also reported by other authors [30,31,32]. Hence, it is concluded that the fluctuations observed in the food and water intake did not cause any treatment-related effects in the TS.

In the subacute female group, there was a reduction in ROW for the stomach (0.14 g/kg BW group) and spleen in the 0.14 and 0.29 g/kg BW dosage groups. There was no clear dose-relationship in reduction of ROW as the highest dose group did not produce any changes. Further microscopic examination of these two organs did not show any correlation to the decrease in size. As the changes were only observed in the subacute female group and prolonged exposure of six months revealed no effect on any of the organs inspected in either gender, these two observations are not considered treatment-related.

No significant differences were seen in the hematology profile of the 28-day study of the treated groups when compared to the control group. The hematology parameter findings for the subacute and chronic studies were similar to published control data, except for the WBC, HGB, MCHC and PLT counts, where slight differences were found between the two reference intervals [31,33]. After prolonged exposure of the test item over a substantial lifetime of the TS, some changes in the blood investigations were observed, where platelet numbers of males from all the treated groups significantly decreased. A drop in the platelet reading may be a cause of concern as they are primarily involved in blood coagulation and hemostasis [34]. Coffee by itself has been shown to reduce platelet activation resulting in reduced platelet aggregation though platelet aggregate formation was not evaluated in the current study [35]. The decreased platelet levels recorded were lower than the average platelet levels in males when compared to the reference interval by He et al. (2017) but were comparable to the reference values from Delwatta et al. (2018) [31,36]. However, the decrease was not dose-dependent, as the highest dose did not further reduce the platelet reading compared with the lowest dose.

Creatinine, urea, cholesterol, triglyceride and glucose levels were similar to published control data [31,36]. It was noted that there were large differences in the control data range published. Hence, the control group data in this study were used for all statistical analysis as the control group TS were subjected to the same environment as the treated groups. Elevated renal profile (creatinine and urea), total protein and creatine kinase in the male treated groups (0.29 and 1 g/kg BW dosage) and elevation of the liver, renal and lipid profiles in the female treated groups (0.29 and 1 g/kg BW dosage) may indicate the TS early response to the test item. A recent study by Riza & Andina Putri et al. (2019) also found an increase in creatinine levels in rats administered coffee [37]. However, in our current study, no further correlation in liver nor kidney histopathology were apparent. No dose-dependent conclusion can be drawn from the changes observed in the 0.29 g/kg BW group, as the highest dose did not exacerbate the parameters measured. Contrarily, the consumption of coffee has been reported to decrease liver enzymes associated with liver damage and has shown protective effects attributed to its polyphenol content [38,39,40,41,42,43]. There were favorable significant reductions in liver enzymes (ALP and ALT) and lipid profile indicator (triglyceride) in the low male dose group but no trend was evident in the higher dose groups except in the female triglyceride level when given the high dose.

The interactions between the different components present in both coffee and TA and additionally with food may have exerted their effects on the parameters measured in this study. Both coffee and TA are made up of various phytochemicals, such as caffeine and polyphenols, found in coffee, and eurycomanone, a major compound found in TA [1,39,40]. Caffeine has been well studied and is reported to have a high tolerance dose of 400 mg/day in human [44,45,46]. Although eurycomanone has been reported to have low bioavailability in rats [47,48], its pharmacokinetic behavior may be modified when incorporated with coffee. A recent study reported coffee’s potential to alter acetaminophen drug pharmacokinetic profile when consumed together [49]. Due to this multi-component nature and interactions, safety evaluation of herbal incorporated food products is crucial.

In this study, the toxicity was assessed in one mammalian rodent model, whereas regulatory submissions may require the safety tests to be conducted in more than one species, typically in either rats, mice or rabbits and in dogs to compare the effect and rate of severity of the toxicity [50]. Despite this limitation, the study was conducted for three different durations that covers the acute response of the administered product, as well as the repeated exposure. The studies covered a substantial duration of the rodents’ lifetimes. Therefore, conclusions drawn from this study are exhaustive.

Considering an average consumption of 2 cups (2.5 g test item per cup) per person per day, an average adult (70 kg body weight), ingests on average 0.07 g/kg body weight of the test item daily. The calculated human equivalent dose (HED) for 1 g/kg BW in rats is 0.16 g/kg BW in humans. As the calculated HED is twice the predicted average daily consumption dosage, the present study suggests that the highest dose tested in the prolonged toxicity study (1 g/kg BW per day) of *E. longifolia* infused coffee, may be well tolerated for human consumption.

## 5. Conclusions

In conclusion, the highest single dose administration of TA coffee (2 g/kg of body weight) did not show acute oral toxicity in Sprague Dawley rats in the present study. The 28-day subacute toxicity study reported no toxic change in any observed parameters. No dose-dependent nor morphological changes were attributed to the consumption of TA coffee in the 6-month chronic toxicity study and consumption of TA coffee at a dose of 1 g/kg/day in male and female rats was identified as the no observed adverse effect level (NOAEL).

## Figures and Tables

**Table 1:** Hematology values of male and female test systems in the 28-day subacute study.

Sex	Dosage (g/kg BW)	WBC (10 ^3^/µL)	RBC (10 ^6^/µL)	HGB (g/dL)	HCT (%)	MCHC (g/dL)	PLT (10 ^5^/µL)
Male	0.00	5.18 ± 2.01	8.05 ± 0.48	16.40 ± 0.82	49.10 ± 2.53	33.40 ± 0.39	11.17 ± 1.97
	0.14	4.06 ± 2.52	8.44 ± 0.31	16.70 ± 0.74	50.74 ± 2.40	32.92 ± 0.40	10.64 ± 0.90
	0.29	5.40 ± 1.66	8.09 ± 0.55	16.34 ± 0.84	48.62 ± 2.92	33.64 ± 0.53	11.77 ± 1.57
	1.00	4.22 ± 1.43	8.03 ± 0.63	16.36 ± 0.82	48.22 ± 2.91	33.94 ± 0.48	11.48 ± 1.04
Female	0.00	6.52 ± 3.29	6.92 ± 0.19	14.42 ± 0.54	40.46 ± 1.42	35.64 ± 0.46	10.82 ± 0.91
	0.14	4.66 ± 2.84	6.63 ± 0.79	13.50 ± 1.90	38.56 ± 5.04	34.98 ± 0.87	10.21 ± 3.30
	0.29	5.30 ± 1.48	6.76 ± 0.36	14.13 ± 0.34	39.38 ± 1.79	35.90 ± 1.23	9.16 ± 4.68
	1.00	3.00 ± 1.78	6.88 ± 0.57	14.34 ± 0.72	40.38 ± 2.17	35.50 ± 0.32	12.51 ± 1.44

Values expressed as mean ± SD (n = 5 for all groups, except n = 4 for group 0.29 g/kg BW female). No significant changes were found (*p* > 0.05). Body weight, BW; white blood cells, WBC; red blood cells, RBC; hemoglobin, HGB; hematocrit, HCT; mean corpuscular hemoglobin concentration, MCHC; platelets, PLT.

**Table 2:** Hematology values of male and female test systems in the 6-month chronic study.

Sex	Dosage (g/kg BW)	RBC (10 ^6^/µL)	HGB (g/dL)	HCT (%)	PLT (10 ^5^/µL)
Male	0.00 (n = 19)	8.41 ± 0.57	72.99 ±3.76	44.92 ± 2.96	6.87 ± 1.42
	0.14 (n = 20)	8.03 ± 0.92	71.99 ± 8.0	43.39 ± 5.27	5.51 ± 1.12 *
	0.29 (n = 20)	8.10 ± 0.39	73.25 ± 3.03	44.78 ± 2.07	5.57 ± 0.95 *
	1.00 (n = 19)	8.06 ± 0.52	72.99 ± 3.49	44.66 ± 3.26	5.35 ± 0.74 *
Female	0.00 (n = 19)	7.11 ± 0.77	65.02 ± 6.58	39.38 ± 4.25	7.81 ± 1.39
	0.14 (n = 19)	6.92 ± 1.43	63.49 ± 14.44	37.81 ± 7.81	8.70 ± 2.44
	0.29 (n = 18)	7.21 ± 0.35	66.68 ± 2.45	39.76 ± 1.65	7.88 ± 1.06
	1.00 (n = 19)	7.67 ± 0.61	71.41 ± 5.46*	42.79 ± 4.06	7.38 ± 0.93

Values expressed as mean ± SD. BW = body weight. * Significantly different from control group at *p* < 0.05.

**Table 3:** Clinical biochemistry values of male and female test systems in the 6-month chronic study.

Parameters	Male Dosage (g/kg BW)				Female Dosage (g/kg BW)			
	0 (n = 19)	0.14 (n = 20)	0.29 (n = 20)	1.00 (n = 19)	0 (n = 19)	0.14 (n = 19)	0.29 (n = 18)	1.00 (n = 19)
*Liver Function Profile*								
Total protein (g/L)	49.66 ± 12.42	43.80 ± 17.07	60.49 ± 10.67 *	56.97 ± 7.49	63.42 ± 16.47	58.49 ± 5.64	66.94 ± 8.18	72.30 ± 6.11
Albumin (g/L)	34.19 ± 10.72	24.97 ± 9.61 *	37.25 ± 7.25	32.45 ±5.08	39.72 ± 10.60	43.39 ± 5.17	53.53 ± 5.94 *	40.46 ± 2.57
*Enzymes*								
ALP (U/L)	123.32 ± 115.61	46.18 ± 26.25 *	118.39 ± 42.71	88.95 ± 38.77	25.79 ± 32.79	24.34 ± 43.78	33.07 ± 22.50	18.44 ± 42.67
ALT (U/L)	63.69 ± 31.90	34.03 ± 21.37 *	65.75 ± 25.36	50.49 ± 14.19	54.29 ± 15.50	50.29 ± 10.10	68.96 ± 17.57 *	57.94 ± 12.06
AST (U/L)	155.73 ± 54.04	137.55 ± 68.31	194.22 ± 54.69	171.56 ± 36.13	195.82 ± 75.82	205.78 ± 59.05	270.29 ± 50.25*	229.72 ± 47.78
LDH (U/L)	1993.46 ± 904.07	1671.46 ± 772.76	1430 ± 726.58	1690 ± 819.68	2053.36 ± 793.69	2363.14 ± 721.41	2367.04 ± 763.32	2615.74 ± 491.17 *
CK (U/L)	408.07 ± 253.91	392.64 ± 174.86	562.87 ± 342.05	701.64 ± 292.43 *	653.74 ± 422.37	537.59 ± 372.26	682.9 ± 277.82	756.82 ± 443.50
*Renal Profile*								
Creatinine (µmol/L)	47.08 ± 13.48	44.27 ± 22.79	61.77 ± 11.69 *	54.83 ± 6.76	55.31 ± 12.92	56.18 ± 6.51	66.91 ± 8.90 *	59.86 ± 6.85
Urea (mmol/L)	5.88 ± 1.33	5.02 ± 2.18	7.11 ± 1.12 *	7.00 ± 1.15	7.02 ± 1.61	7.10 ± 0.81	8.65 ± 1.91 *	8.17 ± 0.87
Uric Acid (µmol/L)	283.07 ± 140.43	247.44 ± 137.08	286.70 ± 129.19	334.95 ± 131.69	379.23 ± 125.43	328.01 ± 142.28	449.77 ± 161.52	382.73 ± 142.01
*Lipid Profile*								
Cholesterol (mmol/L)	1.59 ± 0.52	3.73 ± 11.12	1.52 ± 0.36	1.2 ± 0.40	1.72 ± 0.50	1.94 ± 0.46	2.28 ± 0.44 *	2.21 ± 0.31 *
Triglyceride (mmol/L)	1.29 ± 0.64	0.58 ± 0.37*	1.06 ± 0.59	0.92 ± 0.36	0.89 ± 0.55	0.69 ± 0.45	0.92 ± 0.55	0.46 ± 0.32 *
Glucose (mmol/L)	5.33 ± 4.38	3.83 ± 2.88	6.12 ± 4.22	6.81 ± 5.45	5.27 ± 4.59	3.34 ± 3.43	5.29 ± 5.98	5.60 ± 4.24
Calcium (mmol/L)	1.91 ± 0.62	1.60 ± 0.60	2.12 ± 0.39	1.64 ± 0.25	1.93 ± 0.57	2.22 ± 0.24	2.62 ± 0.32 *	2.36 ± 0.29 *

Values expressed as mean. BW = body weight. * Significantly different from control group at *p* < 0.05. Alkaline phosphatase, ALP; alanine amino-transferase, ALT; aspartate amino-transferase, AST; lactate dehydrogenase, LDH; creatine kinase, CK.

**Table 4:** Relative organ weights of male and female test systems in the 28-day subacute study.

Parameters	Male Dosage (g/kg BW)				Female Dosage (g/kg BW)			
	0 (n = 5)	0.14 (n = 5)	0.29 (n = 5)	1.00 (n = 5)	0 (n = 5)	0.14 (n = 5)	0.29 (n = 4)	1.00 (n = 5)
BW ^a^	295.00 ± 11.73	285.00 ± 17.68	292.00 ± 27.06	314.00 ± 24.08	200.00 ± 16.20	215.00 ± 21.21	203.75 ± 24.62	199.00 ± 24.08
Lung ^b^	0.41 ± 0.04	0.43 ± 0.06	0.45 ± 0.02	0.43 ± 0.04	0.57 ± 0.11	0.51 ± 0.08	0.57 ± 0.08	0.55 ± 0.12
Heart ^b^	0.35 ± 0.02	0.35 ± 0.02	0.33 ± 0.03	0.32 ± 0.04	0.36 ± 0.05	0.35 ± 0.03	0.34 ± 0.01	0.37 ± 0.05
Spleen ^b^	0.18 ± 0.03	0.15 ± 0.03	0.16 ± 0.02	0.15 ± 0.01	0.23 ± 0.02	0.19 ± 0.02 *	0.19 ± 0.01 *	0.21 ± 0.03
Stomach ^b^	0.47 ± 0.05	0.46 ± 0.04	0.53 ± 0.02	0.51 ± 0.07	0.64 ± 0.03	0.56 ± 0.05 *	0.66 ± 0.02	0.65 ± 0.05
Intestinal tract ^b^	0.49 ± 0.08	0.41 ± 0.05	0.52 ± 0.12	0.47 ± 0.09	0.48 ± 0.20	0.65 ± 0.21	0.57 ± 0.10	0.69 ± 0.10
Liver ^b^	3.09 ± 0.24	3.21 ± 0.41	2.91 ± 0.21	2.89 ± 0.15	3.23 ± 0.14	3.29 ± 0.36	3.25 ± 0.30	3.21 ± 0.46
Kidney R ^b^	0.39 ± 0.02	0.41 ± 0.03	0.37 ± 0.03	0.38 ± 0.03	0.38 ± 0.02	0.36 ± 0.05	0.38 ± 0.04	0.39 ± 0.02
Kidney L ^b^	0.39 ± 0.02	0.40 ± 0.03	0.37 ± 0.02	0.38 ± 0.04	0.38 ± 0.03	0.35 ± 0.04	0.37 ± 0.03	0.38 ± 0.02
Adrenal R ^b^	0.01 ± 0.00	0.01 ± 0.00	0.01 ± 0.01	0.01 ± 0.01	0.01 ± 0.01	0.01 ± 0.00	0.02 ± 0.01	0.01 ± 0.01
Adrenal L ^b^	0.01 ± 0.00	0.01 ± 0.01	0.01 ± 0.00	0.01 ± 0.00	0.02 ± 0.00	0.01 ± 0.00	0.02 ± 0.01	0.01 ± 0.00
Testes R ^b^	0.51 ± 0.05	0.51 ± 0.03	0.49 ± 0.06	0.46 ± 0.05				
Testes L ^b^	0.50 ± 0.09	0.50 ± 0.03	0.48 ± 0.04	0.48 ± 0.04				
Ovary R ^b^					0.03 ± 0.01	0.03 ± 0.01	0.03 ± 0.01	0.04 ± 0.02
Ovary L ^b^					0.04 ± 0.01	0.04 ± 0.01	0.03 ± 0.00	0.04 ± 0.01

Values expressed as mean ± SD (n = 5 for all groups, except n = 4 for group 0.29 g/kg BW female). BW = body weight, R = right, L = left, ^a^ Unit: g; ^b^ Unit: % body weights. * Significantly different from control group at *p* < 0.05.

**Table 5:** Relative organ weights of male and female test systems in the 6-month chronic study.

Parameters	Male Dose (g/kg BW)				Female Dosage (g/kg BW)			
	0 (n = 19)	0.14 (n = 20)	0.29 (n = 20)	1.00 (n = 19)	0 (n = 19)	0.14 (n = 19)	0.29 (n = 18)	1.00 (n = 19)
BW ^a^	578.95 ± 57.82	568.00 ± 50.22	557.75 ± 51.75	543.42 ± 100.90	286.32 ± 41.36	291.84 ± 30.70	281.94 ± 19.56	314.17 ± 26.91
Lung ^b^	0.36 ± 0.09	0.37 ± 0.11	0.38 ± 0.07	0.39 ± 0.15	0.53 ± 0.12	0.51 ± 0.12	0.57 ± 0.08	0.48 ± 0.14
Heart ^b^	0.26 ± 0.02	0.25 ± 0.02	0.25 ± 0.02	0.25 ± 0.05	0.30 ± 0.03	0.30 ± 0.04	0.31 ± 0.03	0.30 ± 0.03
Spleen ^b^	0.13 ± 0.02	0.13 ± 0.03	0.13 ± 0.02	0.14 ± 0.05	0.16 ± 0.02	0.17 ± 0.02	0.18 ± 0.04	0.17 ± 0.02
Stomach ^b^	0.50 ± 0.06	0.46 ± 0.04	0.44 + 0.05	0.49 ± 0.17	0.70 ± 0.09	0.71 ± 0.11	0.74 ± 0.08	0.67 ± 0.09
Intestinal tract ^b^	0.40 ± 0.08	0.36 ± 0.08	0.36 + 0.08	0.41 ± 0.10	0.51 ± 0.17	0.49 ± 0.15	0.51 ± 0.10	0.50 ± 0.12
Liver ^b^	2.77 ± 0.39	2.63 ± 0.29	2.80 ± 0.28	2.70 ± 0.29	3.01 ± 0.26	2.94 ± 0.25	3.01 ± 0.28	2.85 ± 0.24
Kidney R ^b^	0.28 ± 0.02	0.27 ± 0.02	0.27 ± 0.02	0.28 ± 0.04	0.31 ± 0.07	0.31 ± 0.06	0.33 ± 0.05	0.32 ± 0.02
Kidney L ^b^	0.28 ± 0.02	0.27 ± 0.02	0.27 ± 0.03	0.28 ± 0.04	0.32 ± 0.03	0.32 ± 0.04	0.33 ± 0.03	0.32 ± 0.03
Adrenal R ^b^	0.004 ± 0.002	0.004 ± 0.002	0.004 ± 0.002	0.005 ± 0.002	0.009 ± 0.005	0.014 ± 0.015	0.011 ± 0.007	0.015 ± 0.022
Adrenal L ^b^	0.004 ± 0.001	0.005 ± 0.002	0.003 ± 0.001	0.005 ± 0.002	0.010 ± 0.005	0.011 ± 0.006	0.011 ± 0.005	0.011 ± 0.004
Testes R ^b^	0.31 ± 0.04	0.31 ± 0.05	0.33 ± 0.03	0.32 ± 0.07				
Testes L ^b^	0.34 ± 0.06	0.32 ± 0.03	0.33 ± 0.03	0.33 ± 0.09				
Ovary R ^b^					0.02 ± 0.01	0.02 ± 0.01	0.03 ± 0.01	0.02 ± 0.01
Ovary L ^b^					0.03 ± 0.01	0.03 ± 0.01	0.03 ± 0.01	0.02 ± 0.01

Values expressed as mean ± SD. BW = body weight, R = right, L = left, ^a^ Unit: g; ^b^ Unit: % body weights. No significant changes were found (*p* > 0.05).

## Appendix A

**Table A1:** Summary of 14-day acute study findings.

Test System	Dosage (g/kg BW)	Mortality	Clinical Signs of Toxicity	Body Weight (g)			Body Weight Gain (%)	Food Intake (g)		Organs Gross Pathology Findings
				Initial	Week 1	Week 2		Week 1	Week 2	
1	0.005	0/1	NAD	219.87	234.52	246.07	10.60	86.05	69.45	NAD
2	0.050	0/1	NAD	216.90	254.89	292.81	25.90	87.35	112.47	NAD
3	0.300	0/1	NAD	216.94	246.34	278.95	22.20	93.79	113.47	NAD
4	2.000	0/1	NAD	234.14	260.60	274.50	14.70	106.92	82.80	NAD
5	2.000	0/1	NAD	243.15	275.31	291.17	16.50	132.83	76.80	NAD
6	2.000	0/1	NAD	202.05	219.19	222.79	9.31	92.12	62.31	NAD
7	2.000	0/1	NAD	211.36	244.51	250.01	15.50	113.11	76.19	NAD
8	2.000	0/1	NAD	218.59	268.09	253.17	13.70	125.38	67.20	NAD
				221.90 ± 16.71	253.50 ± 22.34	258.30 ± 25.98	13.92 ± 2.78	114.10 ± 15.92	73.06 ± 8.19	

The body weight, food intake and percentage body weight gain values are expressed as mean ± SD of 5 test systems given the 2 g/kg dosage in the acute study. No statistical comparison was made as the method used was fixed dose procedure with emphasis on the acute clinical response and no acute death was observed. BW = body weight, NAD = no abnormality detected.

**Table A2:** Food consumption of male and female test systems in the 28-day subacute study.

Weekly Food Consumption	Male Dosage (g/kg BW)				Female Dosage (g/kg BW)			
	0 (n = 5)	0.14 (n = 5)	0.29 (n = 5)	1.00 (n = 5)	0 (n = 5)	0.14 (n = 5)	0.29 (n = 4)	1.00 (n = 5)
Week 1	117.00 ± 11.51	123.00 ± 17.89	109.00 ± 12.94	117.00 ± 16.43	85.00 ± 7.91	91.00 ± 15.17	108.00 ± 28.64	84.00 ± 11.94
Week 2	134.00 ± 17.10	116.40 ± 14.74	130.00 ± 9.35	135.00 ± 12.75	91.00 ± 6.52	99.00 ± 8.94	109.00 ± 38.63	81.00 ± 14.32
Week 3	160.00 ± 20.92	160.00 ± 12.75	145.00 ± 14.14	157.00 ± 17.18	98.00 ± 13.51	116.00 ± 39.91	111.25 ± 49.56	108.00 ± 33.28
Week 4	173.00 ± 8.37	136.00 ± 30.50	148.00 ± 22.80	162.00 ± 13.04	128.00 ± 21.68	166.00 ± 28.81	147.50 ± 22.17	126.00 ± 27.02

Values expressed as mean ± SD (n = 5 for all groups, except n = 4 for group 0.29 g/kg BW female). BW = body weight. No significant changes were found (*p* > 0.05).
